# Comparative bone histology of two thalattosaurians (Diapsida: Thalattosauria): *Askeptosaurus italicus* from the Alpine Triassic (Middle Triassic) and a Thalattosauroidea indet. from the Carnian of Oregon (Late Triassic)

**DOI:** 10.1186/s13358-023-00277-3

**Published:** 2023-08-16

**Authors:** N. Klein, P. M. Sander, J. Liu, P. Druckenmiller, E. T. Metz, N. P. Kelley, T. M. Scheyer

**Affiliations:** 1 Department of Palaeontology, University of Zurich, Karl Schmid-Strasse 4, 8006 Zurich, Switzerland; 2 Department of Palaeontology, Institute of Geosciences, University of Bonn, Nußallee 8, 53115 Bonn, Germany; 3 School of Resources and Environmental Engineering, Hefei University of Technology, 193 Tunxi Road, Hefei, 230009 China; 4 University of Alaska Museum, 1962 Yukon Dr., Fairbanks, AK 99775 USA; 5 Department of Geosciences, University of Alaska Fairbanks, 1930 Yukon Dr., Fairbanks, AK 99775 USA; 6 Museum of the Rockies, Montana State University, 600 W Kagy Blvd., Bozeman, MT 59717 USA; 7 Department of Earth and Environmental Sciences, Vanderbilt University, Nashville, TN 37240 USA

**Keywords:** Triassic marine reptiles, Microanatomy, Tissue type diversity, Coarse parallel-fibred tissue, Globuli ossei, Life style

## Abstract

Here, we present the first bone histological and microanatomical study of thalattosaurians, an enigmatic group among Triassic marine reptiles. Two taxa of thalattosaurians, the askeptosauroid *Askeptosaurus italicus* and one as yet undescribed thalattosauroid, are examined. Both taxa have a rather different microanatomy, tissue type, and growth pattern. *Askeptosaurus italicus* from the late Anisian middle Besano Formation of the southern Alpine Triassic shows very compact tissue in vertebrae, rib, a gastralium, and femora, and all bones are without medullary cavities. The tissue shows moderate to low vascularization, dominated by highly organized and very coarse parallel-fibred bone, resembling interwoven tissue. Vascularization is dominated by simple longitudinal vascular canals, except for the larger femur of *Askeptosaurus,* where simple vascular canals dominate in a radial arrangement. Growth marks stratify the cortex of femora. The vertebrae and humeri from the undescribed thalattosauroid from the late Carnian of Oregon have primary and secondary cancellous bone, resulting in an overall low bone compactness. Two dorsal vertebral centra show dominantly secondary trabeculae, whereas a caudal vertebral centrum shows much primary trabecular bone, globuli ossei, and cartilage, indicating an earlier ontogenetic stage of the specimens or paedomorphosis. The humeri of the thalattosauroid show large, simple vascular canals that are dominantly radially oriented in a scaffold of woven and loosely organized parallel-fibred tissue. Few of the simple vascular canals are thinly but only incompletely lined by parallel-fibered tissue. In the Oregon material, changes in growth rate are only indicated by changes in vascular organization but no distinct growth marks were identified. The compact bone of *Askeptosaurus* is best comparable to some pachypleurosaurs, whereas its combination of tissue and vascularity is similar to eosauropterygians in general, except for the coarse nature of its parallel-fibred tissue. The cancellous bone of the Oregon thalattosauroid resembles what is documented in ichthyosaurs and plesiosaurs. However, in contrast to these its tissue does not consist of fibro-lamellar bone type. Tissue types of both thalattosaurian taxa indicate rather different growth rates and growth patterns, associated with different life history strategies. The microanatomy reflects different life styles that fit to the different environments in which they had been found (intraplatform basin vs. open marine). Both thalattosaurian taxa differ from each other but in sum also from all other marine reptile taxa studied so far. Thalattosaurian bone histology documents once more that bone histology provides for certain groups (i.e., Triassic Diapsida) only a poor phylogenetic signal and is more influenced by exogenous factors*.* Differences in lifestyle, life history traits, and growth rate and pattern enabled all these Triassic marine reptiles to live contemporaneously in the same habitat managing to avoid substantial competition.

## Introduction

Thalattosauria are a group of Middle to Late Triassic marine reptiles with controversially discussed phylogenetic relationships within Diapsida (Druckenmiller et al., 2020; Scheyer et al., 2017). They are rare faunal components in the western Tethys and eastern Pacific realms but they are very common in the eastern Tethys. Finds from the Southern Hemisphere are lacking. Thalattosaurians lived in a wide array of habitats, from intraplatform basins and nearshore environments to the open sea*.* However, near-shore records are rare, e.g., the Muschelkalk deposits from the Germanic Basin lack them. Thalattosaurians have a small or secondarily closed upper temporal fenestra, elongate neural spines, stout and short limbs, and a long powerful, laterally compressed swimming tail. They can be divided into two major clades, Askeptosauroidea and Thalattosauroidea (summarized in Druckenmiller et al., 2020). The Askeptosauroidea are restricted to the Tethyan realm and show the long snout and piercing teeth of a typical fish eater or are, in the case of *Endennasaurus,* edentulous. Thalattosauroidea are known from the Pacific and western and eastern Tethyan realms. They display a remarkable morphological disparity in their feeding apparatus (i.e., elongation and/or ventral curvature of the premaxilla) and dentition (i.e., pointed teeth and crushing teeth), indicating a specialized diet (Rieppel et al., 2005). The increasing specialization in feeding strategies and the accompanied restricted ecological role may have contributed to the eventual extinction of the group (Druckenmiller et al., 2020), accompanied in a long-term perspective by the loss of intraplatform habitats*.* Compared to other contemporaneous marine reptile clades such as Sauropterygia and Ichthyosauria, thalattosaurians are far less abundant in individual numbers and diversity (except for the Xiaowa Formation in Guizhou, China).

Bone histology provides insights into metabolism and physiology of extinct taxa based on the deposited bone tissues and inferred growth rates (e.g., Bakker, 1980; Buffrénil et al., 2021a; Montes et al., 2007; Padian & Horner, 2004; Padian et al., 2004; Ricqlès, 1983, 1992;). From the preserved growth record, growth patterns and life history strategies can be deduced (e.g., Buffrénil & Castanet, 2000; Griebeler & Klein, 2019; Klein & Griebeler, 2018; Klein et al., 2015; Köhler et al., 2012). Bone microstructure (bone compactness [BC] = trabecular/spongious vs. compact organization) that includes the presence and size of a medullary cavity or region and a remodeling zone as well as vascular density, allow insights on the lifestyle of the organism (e.g., Canoville & Laurin, 2010; Dumont et al., 2013; Houssaye et al., 2010, 2014; Klein et al., 2016; Quemeneur et al., 2013) and its habitat preferences (Ricqlès & Buffrénil, 2001).

Bone histology was so far studied in the following Triassic marine reptile groups: in ichthyosaurs (Houssaye et al., 2014, 2018; Kolb et al., 2011; Nakajima et al., 2014), in a variety of Sauropterygia (Hugi, 2012; Hugi et al., 2011; Klein, 2010; Klein & Griebeler, 2016, 2018; Klein et al., 2015, 2016; Krahl et al., 2013; Sander, 1990, 2021; Sander & Wintrich, 2021; Scheyer & Klein, 2021; Scheyer et al., 2021; Wintrich et al., 2017), and in tany-stropheids (Jaquier & Scheyer, 2017; Spiekmann et al., 2020). The different groups display a very high microanatomical and histological diversity that reflects differences in lifestyle, life history traits, and growth rate, enabling them—notably in the Middle Triassic—to live contemporaneously in the same habitats (e.g., Houssaye et al., 2016; summarized in Buffrénil et al., 2021b; Klein et al., 2016, 2022). This diversity spans from fast growing fibro-lamellar tissue in a primary cancellous periosteal organization in ichthyosaurs (summarized in Sander, 2021), a thick cortex of fast-growing radial fibrolamellar bone in plesiosaurs (summarized in Sander & Wintrich, 2021), and a primary fast-growing spongious tissue in some placodonts to slowly deposited and very compact lamellar-zonal tissue in other placodonts (summarized in Scheyer & Klein, 2021). Eosauropterygians (e.g., pachypleurosaurs and nothosaurs) usually have rather compact bone and tissue types that can be summarized as lamellar-zonal (summarized in Klein & Surmik, 2021; Scheyer et al., 2021). Despite the overall high diversity in bone microstructure and tissue types observed across groups, within group variability differs substantially. Ichthyosaurs and plesiosaurs show a rather uniform histology, whereas in Triassic Sauropterygia, habitat and environmental conditions have a high influence on microanatomy and histology (summarized in Scheyer & Klein, 2021; Scheyer et al., 2021; Klein et al., in 2016, 2022), resulting in a certain histological and microanatomical variety. When compared to modern reptiles (Castanet, 2000), most Mesozoic marine reptiles generally show higher growth rates, as is also described for marine mammals (White, 2011). Although Ichthyosauria, Plesiosauria, Pachypleurosauria, Nothosauria, and Placodontia can be roughly distinguished at the group level by the combination of microanatomical and histological data, neither are independent indicators for phylogenetic relationships alone, as was pointed out before (e.g., Ricqlès et al., 2008). Thalattosaurians are the only major Triassic marine reptile group that has not been studied histologically so far.

In the focus of our study are the thalattosaurians *Askeptosaurus*, a basal Askeptosauroidea (e.g., Druckenmiller et al., 2020), and a yet unnamed thalattosauroid (Metz, 2019). *Askeptosaurus* was first erected by Nopsca (1925), investigated by Kuhn (1952) and Kuhn-Schnyder (1960, 1971), and later revised by Müller (2002, 2005). It has a long, straight, and bluntly terminating snout and a strong swimming tail making up more than half of its body length. *Askeptosaurus* could reach body sizes of about 3 m. Three more thalattosaurians have been described and named from the western Tethys: the thalattosauroids *Hescheleria ruebeli* (Rieppel et al., 2005) and *Clarazia schinzi* (Rieppel, 1987), both from the same strata and locality as *Askeptosaurus*, and the askeptosauroid *Endennasaurus acutirostris* from the Norian of Italy (Zorzino Limestone in Lombardia) (Müller et al., 2005). *Askeptosaurus* is restricted to the middle Besano Formation (late Anisian) of the famous UNESCO world heritage locality Monte San Giorgio, Switzerland/Italy. The Besano Formation was deposited in an intraplatform basin, which had stagnant and calm water (Furrer, 2003). Between the continent and the Monte San Giorgio basin, a lagoon was situated, while the Tethys Ocean was in the south (Furrer, 1995).

The thalattosaurian material from the Brisbois Member of the Vester Formation in central Oregon, USA, was found in a nodule containing—besides other faunal elements—mainly disarticulated remains of a minimum of seven conspecific thalattosaurian individuals. The material was described in a Master’s thesis (Metz, 2019), with a publication being currently finalized elsewhere. The Brisbois Member represents a heterogenous marine sequence in the Vester Formation, which was deposited in a collisional marine foredeep basin between two island arcs off the west coast of North America (Metz, 2019). The biostratigraphy of the nodule was determined based on the association of the thalattosaurian material with specimens of the ammonoid *Tropites* sp., indicating a late Carnian age. Based on a wide suite of cranial and postcranial morphological features, the thalattosaurian material from the Brisbois Member represents a new taxon, provisionally regarded as a basal member of the Thalattosauroidea (Metz, 2019). The total body length of the species is estimated at about 4–5 m.

## Aim

Here we present novel data on bone histology and microanatomy of thalattosaurians. The histology of femora, tail vertebrae, a rib, and a gastralium of *Askeptosaurus italicus* from the Besano Formation (late Anisian) of two localities at Monte San Giorgio (Switzerland) and humeri and vertebrae of a thalattosauroid from the late Carnian Brisbois Member of the Vester Formation (Oregon, USA) were studied. The sample of *Askeptosaurus* includes two different-sized individuals.

## Materials

Two tail vertebrae, a rib, three femora, and one gastralium were sampled from two thalattosaurian specimens (Table 1) from two localities in the middle Besano Formation (late Anisian) at Monte San Giorgio assigned to *Askeptosaurus italicus* (PIMUZ T 4839 from Cava Tre Fontane and PIMUZ T 4840 from Valle Stelle, Minerale del Sasso 1) (Müller, 2002, 2005). Both specimens are incomplete, each consisting of the posterior trunk region (i.e., posterior dorsals and ribs, sacral region and hindlimbs) and a fairly complete, characteristically long and powerful tail. The preserved part of PIMUZ T 4839 measures about 2 m (femur length 12 cm), and that of PIMUZ T 4840 about 1.4 m (femur length 8.5 cm). PIMUZ T 4839_vert_a is a caudal vertebra from the middle part of the tail, whereas PIMUZ T 4839_vert_b is a caudal from a more posterior position in the tail. Both vertebrae were cut transversely. The rib sample (PIMUZ T 4839) is from the proximal part of a posterior dorsal rib. The sampled gastralium (PIMUZ T 4840) is a lateral gastral element from the posterior trunk region. The rib and the gastralium were cut perpendicularly to their long axis. The three femora (PIMUZ T 4839, PIMUZ T 4840) were sampled at midshaft. The vertebrae are heavily affected by sediment compaction in mediolateral direction, whereas the femora, the rib, and the gastralium are less compacted.

**Table 1:** Material included in this study. The material of *Askeptosaurus italicus* is from the locality of Monte San Giorgio (middle Besano Formation, late Anisian). The material of the thalattosauroid (Thalattosauria indet.) from Oregon, USA, comes from the Brisbois Member of the Vester Formation and is of late Carnian age

Taxon	Bone	Comment
*Askeptosaurus italicus*		
PIMUZ T 4839 (large individual)	Right femur 12 cm long	Cross section
	Left femur not measured because incompletely preserved	Cross section
	Centrum of anterior tail vertebra (PIMUZ T 4839_vert_a)	Transverse section centrum very compacted
	Centrum of posterior tail vertebra (PIMUZ T 4839_vert_b)	Transverse section centrum very compacted
	Rib	Cross section
PIMUZ T 4840 (small individual)	Right femur 8.5 cm	Cross section
	Gastralium	Cross section
Thalattosauria indet.		
MNCH F71616	Middle dorsal centrum	Transverse section
	2.1 cm length, 2.85 cm height, 3.28 cm width	
MNCH F70662	Posterior dorsal centrum	Sagittal section
	1.8 cm length, 2.55 cm height, 3.1 cm width	
MNCH F64330	Middle caudal centrum	Sagittal section Figure 2.16 in Metz, 2019
	1.91 cm length, 3.1 cm height, 3.11 cm width	
MNCH F64316	Left humerus 8.1 cm long	Core sample Transverse section Figure 2.18 in Metz, 2019
MNCH F64270	Humerus 7.1 cm long	Core sample Longitudinal section/cut obliqually

From the Brisbois thalattosauroid, one mid-dorsal centrum (MNCH F71616), one posterior dorsal centrum (MNCH F70662), and one mid-caudal centrum (MNCH F64330, Fig. 2.16 in Metz, 2019), as well as two humeri (MNCH F64270 and MNCH F64316, Fig. 2.18 in Metz, 2019) were sampled. The centra are not among the largest centra known (compare Metz, 2019: Table 2.4 with Table 1 this study). The two sampled humeri are 8.1 and 7.1 cm long, which is with 101% somewhat longer and with ~ 90% somewhat smaller than another known humerus (MNCH F64309, at 7.9 cm). The morphology of this material was studied by Metz (2019). The material is three-dimensionally preserved and shows little crushing. An overview of the sampled thalattosaurian material and measurements are given in Table 1.

## Methods

Due to the preservation of fossils in the strongly compacted black shales of Monte San Giorgio, the specimens are rather flattened and bones to be sampled needed to be encapsulated and thus stabilized with Technovit ^®^3040 (^©^ Heraeus-Kulzer). Then, a slice was cut out with a Dremel tool with a diamond-studded blade. After extraction, vertebrae were cut transversely and long bones perpendicular to their long axis at midshaft. Because the tissue of the two *Askeptosaurus italicus* specimens is affected by diagenesis and thus relatively poorly preserved, from each femur, two or three thin sections were made from around the midshaft region and were ground to different thicknesses. Thicker thin sections reveal a better growth record, whereas in thinner sections, tissues are easier to assess.

From the Oregon thalattosauroid, only one section was produced from each of the three centra. The middle dorsal centrum was cut transversely, and the posterior dorsal and middle caudal centrum were cut sagittally. The humeri were sampled by core drilling (Sander 2000) across the entire midshaft, from dorsal to ventral, thus sampling the greater part of the midshaft cross section without compromising shape information. The core of MNCH F64316 then was cut to provide a partial transverse section of the bone at midshaft, and the other core (MNCH F64270) to provide a partial longitudinal section of the midshaft region of the bone. All sections were processed into petrographic thin sections using standard methods (Klein & Sander, 2007). Histological terminology follows Francillon-Vieillot et al. (1990). The thin sections were studied under a Leica DM2500LP polarizing microscope. Digital photomicrographs were taken with a Zeiss Axio Imager A1 petrographic microscope equipped with a Panasonic Lumix DMC‐G70 camera in the Institute of Geosciences, Department of Mineralogy, University of Bonn, and a Leica DM 2500 M compound microscope equipped with a Leica DFC 420 digital camera. Overview images were obtained by scanning the thin sections with an Epson V740 PRO high-resolution scanner.

## Histological description

### *Askeptosaurus italicus*

#### Vertebrae

**Fig. 1:**
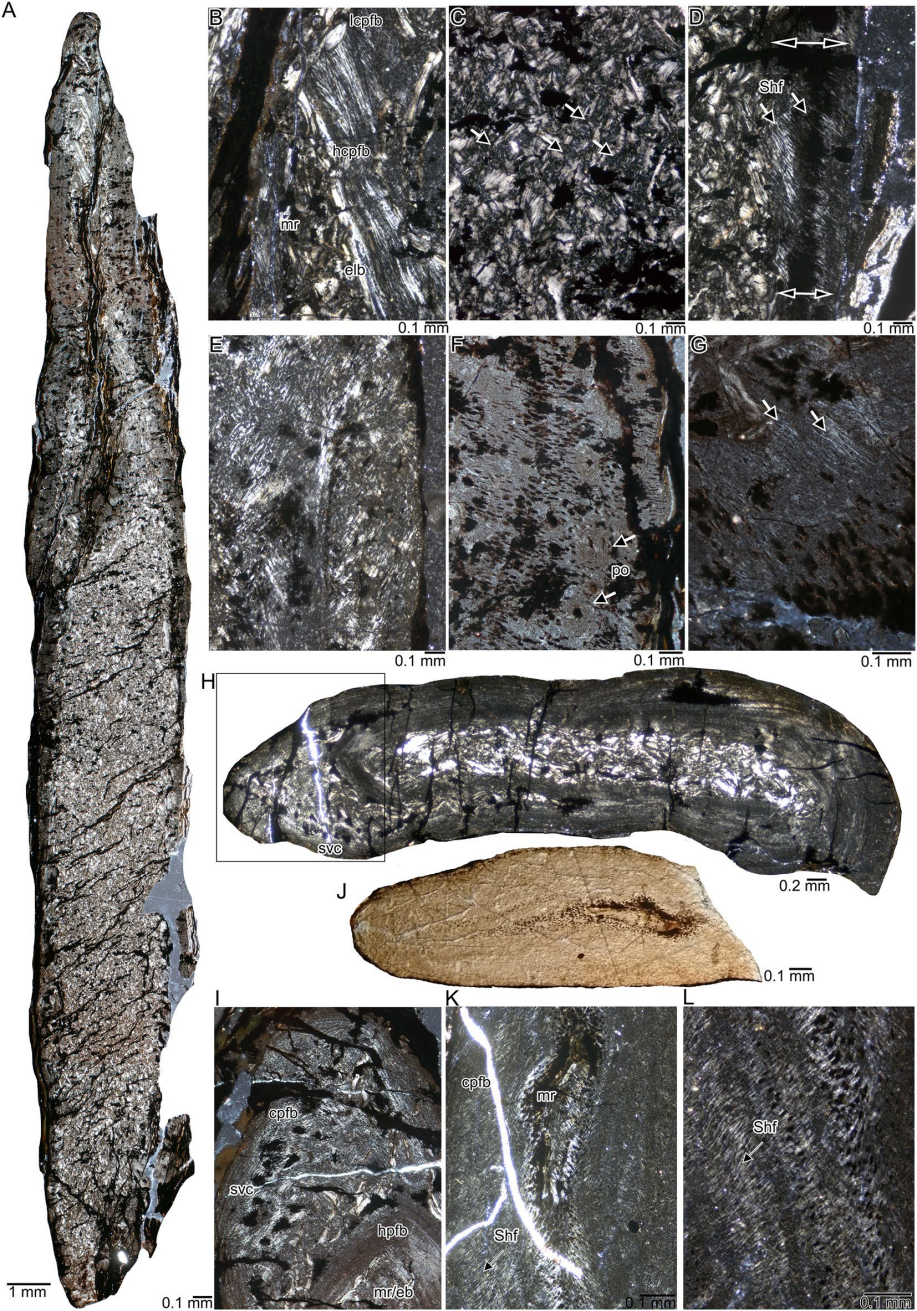
Histology of vertebra (**A**–**G**), rib (**H**,** J**) and gastralium (**I**,** K**,** L**) of *Askeptosaurus italicus*. **A**, Transverse section of laterally compressed tail vertebra (PIMUZ T 4839_vert_a) in polarized light. **B**, Detail of neural spine displaying a medullary region filled by endosteal lamellar bone and surrounded by well and poorly organized coarse parallel-fibered bone of the periosteal region in polarized light. **C**, Collapsed trabecular structure of the inner centrum (PIMUZ T 4839_vert_a) consisting of endosteal lamellar bone (white/bright areas) and patches of calcified cartilage (some marked by arrows) in polarized light. The dark black spots are diagenetically initiated opaque mineral phases. **D**, Detail of medullary region and cortex of the tail vertebra (PIMUZ T 4839_vert_a) in polarized light. Dimension/extension of thin periosteal cortex indicated by double headed arrows. Single headed arrows indicate direction of Sharpey’s fibers (in bright grey/whitish and black, the latter are overgrown by opaque mineral phases). **E**, Detail of periosteal cortex of the tail vertebrae (PIMUZ T 4839_vert_a) in polarized light depicting coarse parallel-fibred tissue showing mainly in the upper left half a honeycomb structure of the thick and coarse scaffold of the parallel-fibred bone. **F**, Detail of periosteal cortex with primary osteons (arrows) of the tail vertebrae (PIMUZ T 4839_vert_a) in polarized light. **G**, High magnification of the periosteal cortex of the tail vertebra (PIMUZ T 4839_vert_a) with higher organized and less coarse parallel-fibred tissue in polarized light. The arrow marks Sharpey’s fibers. **H**, Cross section of the rib of PIMUZ T 4839 in polarized light. Note the dense central medullary region filled by endosteal bone and the nearly avascular, compact periosteal cortex consisting of coarse parallel-fibred bone. **I**, Cross section of the gastralium of PIMUZ T 4840 in normal light. Note the compact avascular tissue and the accumulations of osteocyte lacunae. **J**, Detail of rib tissue (endosteal and periosteal) in polarized light as marked with the rectangle in H). Note the coarse parallel-fibred tissue and the simple vascular canals obscured by opaque mineral phases (in black). **K**, Detail of gastral tissue of PIMUZ T 4840 in polarized light. Note the different degree of organization of the coarse parallel-fibred tissue in polarized light. The arrow marks the direction of Sharpey’s fibers. **L**, Enlargement of tissue of the gastralium in polarized light. Note the coarse parallel-fibred tissue and Sharpey’s fibers and the dense accumulations of osteocytes. The arrow marks the direction of Sharpey’s fibers. Abbreviations: *eb*, endosteal lamellar bone; *ipo*, incompletely lined primary osteons, *hclp* honeycomb-like pattern, *hpfb* highly organized coarse parallel-fibred bone, *ppfb* poorly organized coarse parallel-fibred bone, *mr* medullary region, *Shf* Sharpey’s fibers, *svc* simple vascular canals

The transverse sections of the two sampled caudal vertebrae of *Askeptosaurus italicus* (PIMUZ T 4839_vert_a &_b) are highly laterally compressed. The original morphology of the centra is collapsed, resulting in a thin, elongated rod-like shape of the vertebral sections (Fig. 1A). In addition, numerous large parallel and smaller intercalated cracks run in a roughly lateromedial direction across the entire vertebrae. Those cracks are widened and filled by an opaque (i.e., black) mineral phase (Fig. 1A–G). Furthermore, isolated patches of opaque mineral growth also occur in the periosteal and endosteal tissue (Fig. 1A–G). The transition (i.e., neurocentral suture) between centrum and neural arch cannot be identified. The heavily compressed centrum still allows the identification of a rather thin compacta that encompasses a formerly spongious area. that The spongious region consists of (now collapsed) secondary trabeculae made of lamellar bone permeated by rare local patches of calcified cartilage (Fig. 1C). The slender neural spine is compressed as well, but the original, more compact structure is better preserved here than the spongious structure of the centrum (Fig. 1B). A rhombic medullary region filled by endosteal bone is surrounded by an avascular and highly organized coarse parallel-fibred tissue (Fig. 1A, B). The rest of the compact cortex consists of a poorly vascularized to avascular coarse parallel-fibred tissue that is locally less (Fig. 1E) or more strongly organized (Fig. 1G). The collagenous scaffolding of the parallel-fibred bone is so coarse and thick that it locally forms a honeycomb-like pattern due to the crossing of the single thick collagen fibers (Fig. 1E). Locally, Sharpey’s fibers enter the cortex at high angles, and the opaque mineral phase basically follows this arrangement (Fig. 1D, F, G) (see Konietzko-Meier & Sander, 2013 for similar diagenetically affected fibers). Only very few small, mainly longitudinal primary osteons (Fig. 1F) and simple longitudinal vascular canals are identified. The latter as well as osteocyte lacunae are largely obscured by the opaque mineral phase (Fig. 1F, G). The cortex is in general less organized in PIMUZ T 4839_vert_b when compared to PIMUZ T 4839_vert_a. However, the latter shows a lesser amount of calcified cartilage. Due to the heavily compacted shape of the vertebrae, overall vertebral compactness cannot be estimated. In the outer cortical layer, bone compactness is nearly 100%.

#### Rib

The rib sample (PIMUZ T 4839) is also compressed and has an oblong oval cross section (Fig. 1H). Patches of opaque mineral phases are scattered across the section (Fig. 1H). Cracks running through the section are enlarged by the opaque mineral phase (Fig. 1H, J). The centrally located medullary region is filled by dense endosteal bone and small patches of calcified cartilage. The region is now very compact; if it was originally more cancellous remains unclear (Fig. 1J). The periosteal tissue is also very compact and consists of poorly organized parallel-fibred tissue in the inner cortex and more highly organized parallel-fibred tissue in the outer half (Fig. 1J). The tissue is made of a thick and coarse collagenous scaffolding of the parallel-fibred tissue, locally resulting in a honeycomb-like pattern as in the vertebrae. The high degree of the organization of tissue implies a stratification of the tissue (Fig. 1H, J), resulting from cyclical apposition. Osteocyte lacunae are small and flat. One side of the cross section is made of poorly organized, coarse parallel-fibred tissue, Sharpey’s fibers, one primary osteon, and few simple longitudinal vascular canals (Fig. 1J, I). Towards the inner cortex, i.e., the endosteal region, this fast-growing tissue is separated from the endosteal region by a thick layer of avascular and highly organized tissue (Fig. 1H, I). The rib is mostly compact (99.91%), because only very few vascular canals occur along the posterior side, and the medullary region is filled with secondary endosteal bone.

#### Gastralium

The gastralium (PIMUZ T 4840) has an elongated oval cross section—maybe the result of some crushing—and is compact. The center of the gastralium contains an accumulation of large osteocyte lacunae that surround an area of highly organized, coarse parallel-fibred tissue (Fig. 1K, L). This area is surrounded by numerous thick round osteocyte lacunae that appear diagenetically enlarged by an opaque mineral phase (Fig. 1K). The rest of the tissue is poorly organized parallel-fibred tissue containing smaller and fewer osteocyte lacunae that are arranged in three main layers (Fig. 1K, L). The gastralium has a compactness of 100% due to the lack of any vascular canals or other cavities. Please note that it is difficult to calculate compactness precisely due to widening of and around those osteocyte lacunae by opaque mineral growth.

#### Femora

**Fig. 2:**
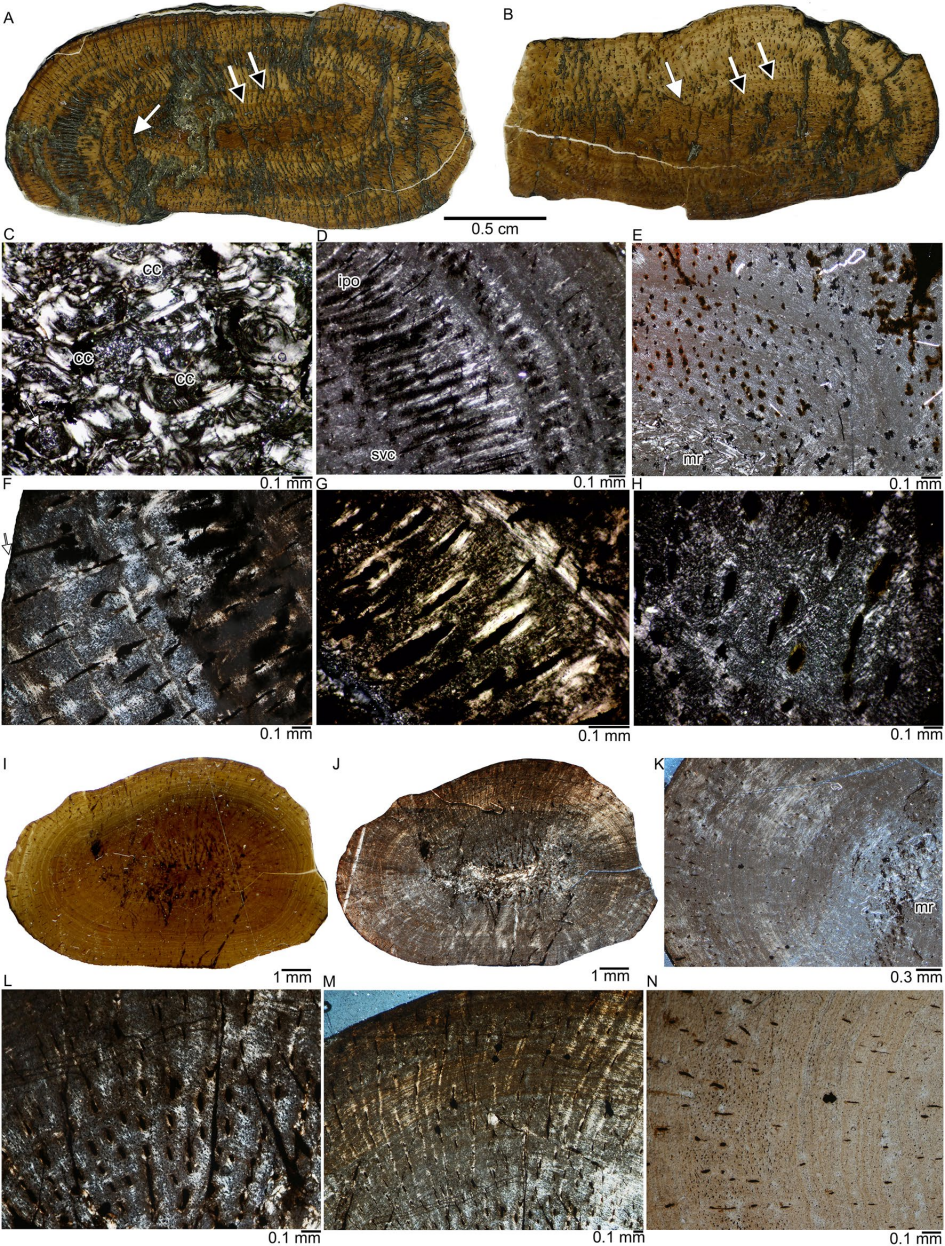
Histology of femora of *Askeptosaurus italicus*. **A**, Midshaft cross section of the left femur of PIMUZ T 4839 in normal light. Note the overall radial organization of vascular canals and different growth phases as indicated by distinct lines of arrested growth and/or changes in color. Note the shifts of the growth layers indicating compaction (white arrows). Black arrows indicate LAGs. **B**, Midshaft cross section of the right femur of PIMUZ T 4839 in normal light. Vascularity is less radial and more longitudinally organized*.* Note the shifts of the growth layers indicating compaction (white arrows). Black arrows indicate LAGs. **C**, Patches of calcified cartilage embedded in endosteal lamellar bone in the medullary region of the right femur. **D**, Strong radial organization of simple vascular canals and incompletely lined primary osteons in the outer cortex of the left femur. Note the coarse and loosely organized parallel-fibred tissue alternating with more with more higher organized and more finely organized parallel-fibred tissue. **E**, Detail of cortex of the right femur depicting alternating coarse and loosely organized parallel-fibred tissue and with more higher organized and more finely organized parallel-fibred tissue. **F**, Enlargement of outer cortex of the left femur. Note the simple vascular canal (white arrow) that opens into the surface and the coarse nature of the parallel-fibred tissue. **G**, Region with primary osteons that are completely and incompletely lined by parallel-fibred bone in the left femur. **H**, Enlargement of coarse parallel-fibred tissue with simple vascular canals. **I**, Midshaft cross section of the left femur of PIMUZ T 4840 in normal and **J**, in polarized light. Note the principally radial orientation of the mainly short radial and longitudinal vascular canals. Vascular density is lower when compared to the femora of PIMUZ T 4839. **K**, Detail of the cortex from the postaxial bone side in polarized light. Note that the outer cortex shows more vascular canals than the inner cortex. The entire cortex is stratified by growth marks. **L**, Detail of well vascularized (by simple vascular canals) and poorly organized inner tissue visible in I and J dorsally to the medullary region in polarized light. Note the coarse nature of the loosely organized parallel-fibred tissue. **M**, Radially organized vascular canal system at the dorsal bone side. Note the funnel-shaped arrangement of tissue along the simple radial vascular canals in polarized light. **N**, Detail of cortex depicted in K in normal light. Note the accumulation of osteocyte lacunae in the left part of the picture. Abbreviations: *cc*, calcified cartilage; *ipo*, incompletely lined primary osteons; *mr*, medullary region; *svc*, simple vascular canals

The left and right femur samples of PIMUZ T 4839 show indication of compaction, although they are not as strongly affected as the vertebrae. Both femora document breakage of tissue, mainly indicated by a displacement of growth layers (Fig. 2A, B, white arrows). In addition, both have an elongated, oval cross section (Fig. 2A, B). The femoral sample of PIMUZ T 4840 shows only slight compaction and has a round oval cross section (Fig. 2I). All three femoral samples are very compact without a free medullary cavity (Fig. 2A, B, I, J). They all share a small central medullary region, which is slightly compacted, but there is no indication of an originally large cavity (Fig. 2C).

At midshaft, the medullary region contains endosteal bone and some local patches of calcified cartilage (Fig. 2C). Sections more distally or proximally in relation to the midshaft show a higher amount of calcified cartilage. PIMUZ T 4840 also has diagenetic infilling (but no obvious enlargement) of osteocyte lacunae and vascular canals by an opaque mineral phase, as well as few square-shaped mineral patches (i.e., cubic crystals, probably pyrite) scattered over the tissue. As in the vertebrae and the rib of the same individual, the femur sections of PIMUZ T 4839 are more affected by mineral growth than PIMUZ T 4840 (Fig. 2A, B). The matrix in all femur samples is dominated by very coarse parallel-fibred tissue that can be more or less organized and consists of a thick and coarse collagenous scaffolding of the parallel-fibred tissue that forms locally a honeycomb-like pattern (Fig. 2F–H). In the smaller femur (PIMUZ T 4840), the tissue fibers are less coarse (Fig. 2K–M). Osteocyte lacunae are numerous but small (if not widened by the opaque mineral phase) (Fig. 1N).

Simple vascular canals dominate, and only very few show the beginning of a centripetal lining as in primary osteons. However, the tissue that started to line the canals is parallel-fibered and not lamellar bone (Fig. 2D, F, G, H), the usual case in primary osteons (Francillon-Vieillot et al., 1990). The dominant vascular canal orientation in the femora is radial at midshaft (Fig. 2A) in PIMUZ T 4839 and radial and longitudinal in PIMUZ T 4840. Except for the dorsal bone side, the radial canal orientation is, however, less obvious in the smaller femur sample (PIMUZ T 4840) (Fig. 2I–M). Locally, funnel-shaped simple radial canals occur (Fig. 2M), which are also known from some nothosaurs (Klein, 2010; Klein et al., 2016). Due to the compacted medullary region and low vascular density in the smaller femur (PIMUZ T 4840, BC = 97.8%) and the moderate vascular density in the two larger femora of PIMUZ T 4839 (BC =  ~ 80%), bone compactness is rather high in all femora.

All three femur samples show growth marks. In the two femora of PIMUZ T 4839, growth marks appear in form of lines of arrested growth (LAGs) that are accompanied each by additional closely spaced rest lines. A further separation into zones and annuli is only partially possible (Fig. 2). Both samples of PIMUZ T 4839 show between 6 and 8 LAGs, depending on bone side. The left and right femur share two distinct LAGs spacing the same distance apart in the inner cortex (Fig. 2A, B; black arrows). The innermost cycle is close to the medullary region, the 2nd cycle is also in the inner cortex, whereas the 3rd cycle is the widest, forming most of the middle cortex. The rest of the LAGs are deposited at a regular spacing in the outer cortex. As is to be expected, the growth record of the left and right femur match. Please note that the growth marks are shifted against each other due to compaction (Fig. 2A, B, arrows).

In PIMUZ T 4840, a growth mark count is not possible. Distinct LAGs are absent, only alternating layers of less organized (i.e., zones) and more organized tissue (i.e., annuli) can be distinguished. However, these do not alternate, and the more highly organized layers which show additional stratification by highly organized tissue, are more numerous (Fig. 2K, M, N). Thus, identification of an annual growth mark sequence is difficult. The femur of PIMUZ T 4840 shows in the innermost cortex of its ventral bones side a well-vascularized (simple longitudinal vascular canals) and loosely organized coarse parallel-fibred bone (Fig. 2L). This tissue indicates faster growth during an earlier ontogenetic stage, and it is here interpreted as the tissue laid down during the juvenile stage. Interestingly, the rest of the tissue of PIMUZ T 4840 indicates lower growth rates (i.e., is more organized and less vascularized) for the rest of the cortex when compared to PIMUZ T 4839, despite being smaller. PIMUZ T 4839, on the other hand, lacks any indication of a juvenile tissue. None of the femur samples show remodeling or secondary osteons.

The smaller PIMUZ T 4840 and the larger PIMUZ T 4839 do not resemble an ontogenetic series as expected. Instead, the differences may be due to a random intraspecific variability, sexual dimorphism (as was already suggested by Müller, 2002 on morphological grounds for *Askeptosaurus italicus*) or taxonomic differences, or all of this.

### Thalattosauroidea indet

#### Vertebrae

**Fig. 3:**
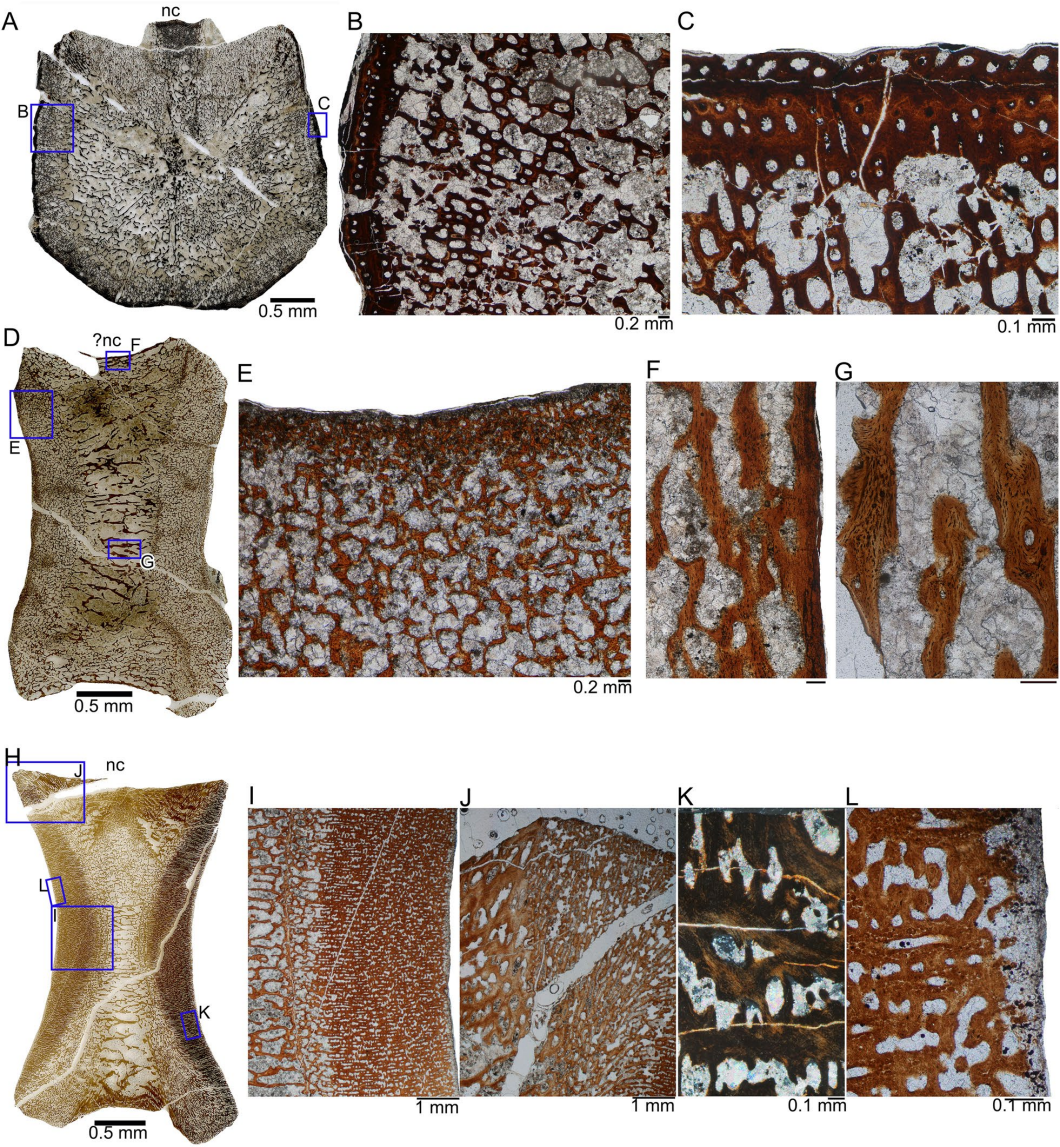
Histology of vertebral centra of the thalattosauroid from Oregon. **A**, Transverse section of a middle dorsal centrum (MNCH F71616) in normal light. Note the overall spongious structure and very thin cortex surrounding the centrum. Rectangles mark the enlarged area depicted in **B** and **C**. **B**, Detail of the thin outer cortex and the spongious inner structure of MNCH F71616 in normal light. **C**, Enlargement of the thin cortex of MNCH F71616 in normal light. **D**, Sagittal section of a posterior dorsal centrum (MNCH F70662) in normal light*.* Rectangles mark the enlarged area depicted in** E**,** F**, and** G**. **E**, Detail of the anterior/posterior margin of MNCH F70662 depicting collapsed trabeculae and a matrix of cartilage (greyish–blackish) in normal light. **F**, Primary trabeculae reaching into the outer cortex in MNCH F70662. **G**, Detail of primary trabeculae in MNCH F70662 in normal light. **H**, Sagittal section of a middle caudal centrum (MNCH F64330) in normal light. Rectangles mark the enlarged area depicted in **I**–**L**. **I**, Anterior or posterior bone side depicting a spongious structure in the inner and a more compact area towards the outer cortex of the middle caudal centrum MNCH F64330 in normal light. **J**, Boundary between the periosteal tissue to the left and the endochondral, much more finely structured tissue to the right in MNCH F64330 in normal light. **K**, Detail of parallel-fibred periosteal tissue in the centrum MNCH F64330 in polarized light. **L**, Detail of the anterior/posterior margin depicting globuli ossei and a cartilage matrix below the articular surface of MNCH F64330 in normal light. Abbreviation: *nc* neural canal

The transverse section of the mid-dorsal centrum MNCH F71616 corresponds largely to the periosteal cortex, because the sample was taken at the center of growth of the centrum, where the endochondral domain is smallest (Fig. 3A). The section shows an overall spongious tissue, which is, however, not homogenous but is divided into different areas by their grade of tightness, i.e., by larger and smaller secondary and few remaining primary trabeculae and differently sized and formed intertrabecular spaces. From the center of the centrum, two lateral cones reach dorsally, which are divided by an additional smaller cone in their middle that has thicker trabeculae (Fig. 3A). The two lateral cones are formed by the growing surfaces of the neurocentral suture (Fig. 3A), i.e., they are the traces of the attachment of the neural arch. The smaller cone in the center is the trace of the growing floor of the neural canal. The center of the centrum, in elongation of the middle cone, shows thicker trabeculae and smaller intertrabecular spaces as well. The center is surrounded by an area of thin trabeculae and large, irregularly shaped intertrabecular spaces as a result of erosion and formation of secondary trabeculae. The general orientation of these spaces is radial (Fig. 3A). This area forms most of the transverse section.

Outwards from this central region follows a ring of primary cortex, which is less spongious due to smaller primary intertrabecular spaces that are mainly round or oval, representing here largely longitudinal simple vascular canals of which most are already enlarged by erosion. These primary canals are arranged in radial rows, and the secondary trabeculae are also predominantly radial. The outer cortex is relatively compact with longitudinal simple vascular canals and primary osteons occurring that are arranged in circumferential rows (Fig. 3B, C). Vascular density is moderate to high. The cortex mainly consists of parallel-fibred tissue that is deposited in different degrees of organization (Fig. 3B, C). Growth marks are not visible. The compactness of the entire transverse section is 38.5%. The compactness of the outermost primary cortex (not yet affected by erosion) at the centrum’s margin is ~ 84.6%.

The sagittal section of the posterior dorsal centrum (MNCH F70662) is in general more spongious when compared to the sagittal section of the caudal centrum (MNCH F64330) and does not show any compact areas (Fig. 3D, E). However, its trabecular architecture is consistent with the middle dorsal centrum cut in transverse section (MNCH F71616). The entire structure is highly disturbed, showing in most areas broken and incomplete or interrupted trabeculae (Fig. 3E). All trabeculae consist of lamellar bone containing numerous flat osteocyte lacunae (Fig. 3F, G). The trabeculae in the inner region are secondary but towards the outer margins, trabeculae are still primary (Fig. 3G). Towards the margins including the anterior and posterior articular surfaces, where the trabecular structure is denser, trabeculae are embedded in a cartilage matrix (Fig. 3E), as is to be expected in the endochondral domain. The periosteal bone of the floor of the neural canal and the ventral side of the centrum consists of a very thin primary cortex of parallel-fibred tissue with numerous osteocyte lacunae (Fig. 3F). The sagittal section of the posterior dorsal centrum MNCH F70662 (Fig. 3D) is more rectangular and less amphicoelous than the caudal centrum MNCH F64330 (Fig. 3H) that shows distinct amphicoely. Besides preservational artefacts, the floor of the neural arch and the ventral surface of MNCH F70662 are flat or straight (Fig. 3D). The floor of the neural canal of MNCH F64330 is also nearly straight but its ventral surface is clearly concave (Fig. 3H).

Whereas the microanatomy of the posterior dorsal centrum MNCH F70662 is consistent with that of middle dorsal MNCH F71616 (Fig. 3A, D; considering the different section plane), the microanatomy of caudal centrum F64330 is different (Fig. 3H). Although both, the posterior dorsal and the caudal centrum, are in general spongious, they are divided up differently into areas that vary in organization and compactness. The center of both centra shows large trabeculae made of secondary endosteal (lamellar) bone that are oriented preferentially in anteroposterior direction (Fig. 3A, D). These trabeculae are separated by large, irregular intertrabecular spaces. In the caudal centrum MNCH F64330 (Fig. 3H–L), this part, representing the periosteal cortex, becomes wider towards the dorsal and ventral side, resembling an hour-glass shape because of the vertebra’s amphicoelous shape*.* The orientation of the trabeculae changes here, and intertrabecular spaces abruptly decrease in size and the structure is more compact (Fig. 3H–J). This presumably indicates the transition from the periosteal to the endochondral domain.

In the endochondral domain of MNCH F64330, which is sharply set off from the periosteal cortex (Fig. 3J), the trabeculae contain in their centers a mixture of coarse parallel-fibred bone, calcified cartilage, and globuli ossei (Fig. 3I–L). Towards the anterior and posterior articular surfaces, trabeculae contain globuli ossei surrounded by parallel-fibred bone. In the outermost cortex of the articular surfaces, globuli ossei dominate the matrix, indicating the formation of primary endochondral trabeculae from the hypertrophied cartilage of the articular surface (Fig. 3L) (Buffrénil & Quilhac, 2021; Quilhac et al., 2014; Wintrich et al., 2020). The outermost margin of the articular surface is formed by cartilage (Fig. 3L). The periosteal tissue consists of parallel-fibred bone but no osteocyte lacunae are visible (Fig. 3N). The dorsal and ventral periosteal tissue is interspersed with Sharpey’s fibers. Neither growth marks nor secondary osteons were identified in the three vertebral samples.

Bone compactness differs between the two centra cut in sagittal section. It is 44.8% in the caudal centrum MNCH F64330 to 34.6% in the posterior dorsal centrum MNCH F70662. Compactness of the endochondral domain is relatively constant in the caudal centrum MNCH F64330 at 75–80%, but it varies between 39 and 56% in the dorsal centrum MNCH F70662. Compactness values between the transversely and sagittally sectioned dorsals cannot be compared.

The marked histological differences between the dorsal centra MNCH F71616 and MNCH F70662, on one hand, and the caudal centrum MNCH F64330 on the other, may be related to anatomical position, to different ossification patterns between parts of the vertebral column, to taxonomy, or to ontogeny (although they share a similar size). MNCH F70662 and MNCH F71616 may be ontogenetically older than MNCH F64330.

#### Humeri

**Fig. 4:**
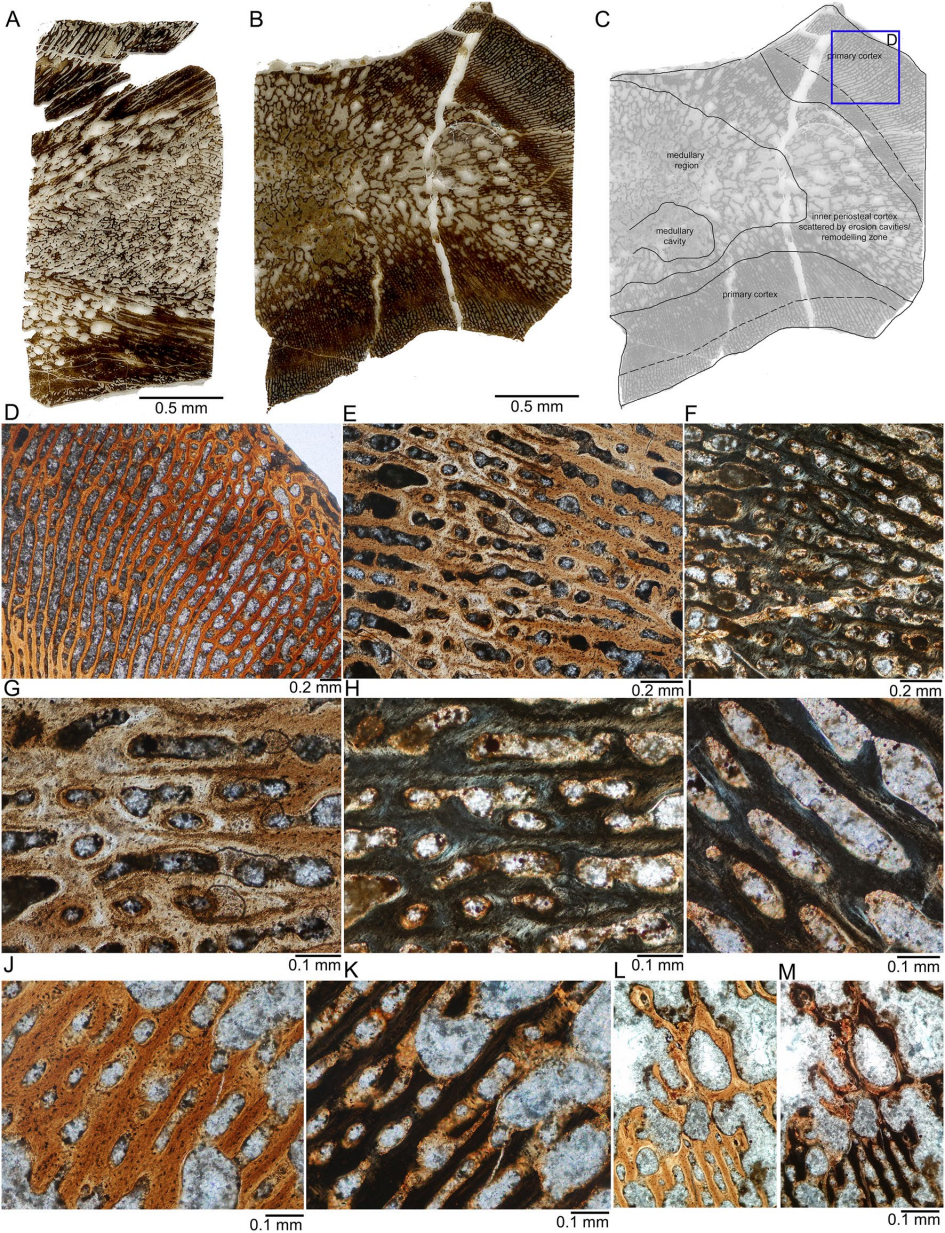
Histology of humeri of the thalattosauroid from Oregon. **A**, Humerus (MNCH F64270), longitudinal section, core sample. Note that the core was cut somewhat off-center, the neutral area is to the left, distal to the right. Image in normal light. **B**, Humerus (MNCH F64316), transverse section in normal light. **C**, Interpretative sketch of the tissue distribution in MNCH F64316. **D**, Detail of periosteal cortex in MNCH F64316 in normal light. Note the radial trabecular structure of the primary periosteal cortex. The middle layer is seen in the lower left corner, but most of the field of view is covered by the outer layer. The outermost layer is in the upper right corner. **E**, Detail of primary periosteal cortex in MNCH F64316 in normal and **F**, polarized light showing the transition zone between the middle (right) and outer layers (left). Note the high amount of plump osteocyte lacunae. Simple vascular and incipient primary osteons canals are mainly oriented longitudinally. **G**, Enlargement of the transition zone between the middle and outer layers in the periosteal cortex of MNCH F64316 in normal and **H**, polarized light. Note the low amount of osteocyte lacunae in a relatively high organized parallel-fibred tissue. Vascularity is high, consisting of large longitudinal to radial simple vascular canals with limited osteonal lining. **I**, Enlargement of parallel-fibred tissue and simple vascular canals in MNCH F64316 in polarized light. **J**, Detail of the middle layer of the periosteal cortex transitioning to the inner layer (upper right) in MNCH F64316 in normal and K, polarized light. The primary parallel-fibred tissue of the middle layer is here less organized between the vascular canals but partially starts to line vascular canals in higher organization. **L**, Detail of secondary endosteal trabeculae (top) and primary periosteal ones (bottom) at the transition from the middle to the inner layer of the periosteal cortex in MNCH F64316 in normal and **M**, polarized light

Two midshaft core samples (MNCH F64316, transverse section; MNCH F64270, longitudinal section) were available for study (Fig. 4). The transverse humerus section, cut from MNCH F64316, exhibits a large medullary region in the center of the humerus that reaches into the pre- and postaxial bone sides (Fig. 4B, C). The medullary region of MNCH F64316 contains a large erosion cavity (filled with sediment) that is located closer to the ventral margin and which is in surrounded by smaller mainly round to oval intertrabecular spaces (Fig. 4L, M). In the inner cortex, the periosteal bone shows scattered round to oval erosion cavities. This remodeling zone is most narrow at the ventral bone side and is thickest towards the postaxial bone side (Fig. 4C). Towards the dorsal side, the outer cortex is damaged, and only the innermost part of the remodeling zone is here preserved (Fig. 4C). The boundary to the periosteal cortex is here either not sampled or obscured by large erosion cavities destroying and dominating the periosteal tissue (Fig. 4B, C).

Most trabeculae in the periosteal cortex are still primary and contain small osteocyte lacunae (Fig. 4L, M). The trabeculae in the medullary region and the remodeling zone enclose irregularly formed and round to oval intertrabecular spaces (Fig. 4B, C). In the center—as far as is visible—the intertrabecular spaces and trabeculae are smaller, but both become larger towards the postaxial side (Fig. 4C).

The periosteal cortex has a primarily spongious, i.e., trabecular, structure. (Fig. 4). The tissue consists of poorly organized parallel-fibred bone and in some areas also of woven bone, containing locally static osteocytes (Fig. 4D–K). Simple vascular canals dominate the tissue, only few show an incomplete and very thin (i.e., initial) lining by parallel-fibred bone (Fig. 4H, I), maybe indicating the beginning of the formation of primary osteons. However, the entire sample does not contain any mature primary osteons (i.e., an osteon completely lined by parallel-fibered or lamellar tissue). In the transverse section, the vascular organization is mainly radial, consisting of large longitudinal and radial simple vascular canals (Fig. 4). This is because the section is placed exactly at midshaft.

The periosteal cortex can be roughly divided into three layers in the cross section (Fig. 4C). This division is mainly based on changes in vascular density and orientation. There are no growth marks of any kind visible, such as lines of arrested growth or annuli. The inner periosteal cortex is largely obscured by scattered erosion cavities. It is, however, easy to distinguish from the two outer layers by a less spongious structure, i.e., the vascular canals are smaller and the bony trabeculae are more massive and thicker (Fig. 4C). The intermediate layer has much smaller vascular canals and thick trabeculae with an apparent higher number of longitudinal canals (Fig. 4D, bottom left). The outer cortex is more spongious than the intermediate one but less spongious than the inner layer. The outer cortex is further characterized by a principally radial vascular canal organization that grades into a more longitudinal organization (Fig. 4D). In the outermost cortex, canal orientation changes also to a more laminar pattern (Fig. 4B, D). The boundary between the middle and outer layer may well represent an annual growth mark, a similar situation is ubiquitous in plesiosaurs (Sander & Wintrich, 2021; Wintrich et al., 2017). The compactness of the entire core of MNCH F64316 is 44.7%; that of the cortex varies between 58 and 86%.

In longitudinal section, cut from the core of MNCH F64270, Fig. 4A), vascular canals fan out from the growth center towards the proximal/distal end of the humerus. Canal orientation thus changes from a fully radial orientation to one that is parallel to the well-marked boundary between the endochondral and periosteal domain (Fig. 4A). Because of the foreshortened morphology of the humeri, this boundary and thus the canal direction is more than 35° from the bone long axis.

## Discussion

### Ontogenetic stage of individuals

#### *Askeptosaurus italicus*

The two sampled *Askeptosaurus* individuals clearly differ in femur size (femur length is 8.5 cm in PIMUZ T 4840 and 12 cm in PIMUZ T 4839), and overall size of the preserved body part, suggestive of PIMUZ T 4839 being a larger animal. The larger specimen (PIMUZ T 4839) is preserved from the mid trunk region to the posterior tail region and measures 200 cm as preserved. Its total body size was thus likely close to the maximal recorded body size of *Askeptosaurus*, which is 3 m (Müller, 2005). The smaller specimen (PIMUZ T 4840) is less complete. The preserved part, from the posterior trunk (sacral) region to the posterior tail region, measures about 140 cm. Even when considering the missing trunk part in PIMUZ T 4840, the specimen was smaller, maybe about 70% of the size of PIMUZ T 4839. However, both individuals have reached more than half of the maximal estimated body size and are quite large. Neither size nor morphology (i.e., degree of ossification) do indicate early ontogenetic stages for either specimens.

The parallel-fibred tissue of the femur of the smaller *Askeptosaurus* specimen is less coarse, more highly organized, and less vascularized when compared to the larger *Askeptosaurus* specimen. In addition, they differ in vascular canals organization: in the smaller specimen, longitudinal vascular canals dominate, whereas the larger specimen has a clear dominance of radial canals. Both individuals show vascular canals that open onto the bone surface and no change in tissue in the outer cortex, which in both indicates continued growth. The smaller *Askeptosaurus* specimen has remains of a tissue indicating an earlier ontogenetic stage in its innermost cortex, and its growth marks are not very distinct. The larger specimen does not show a “juvenile” tissue in its inner cortex and growth marks are distinct in form of lines of arrested growth. In conclusion, based on histology and differences in growth pattern, PIMUZ T 4840 and PIMUZ T 4839 are likely not representing a growth or ontogenetic series, as one might have expected. Differences might be the result of developmental plasticity, sexual dimorphism (i.e., difference in growth between both sexes), or even taxonomy. However, our sample size is too small to further elaborate on this. Müller (2002) found in his morphologically based study two size classes among the *Askeptosaurus* sample he studied and discussed different species and/or sexual dimorphism as possible explanation. However, he also states that none of these interpretations can be corroborated based on the present material (Müller, 2002).

#### Thalattosauroidea indet

As suggested by the relatively small size of the centra and the humeri, which do not represent the largest known elements (Metz, 2019), this material may not represent fully grown individuals. However, the dorsal centra from Oregon presumably represent adult individuals because of the nearly completely secondary nature of the trabeculae in both the periosteal and endochondral domains.

The histology of the caudal centrum and the humeri indicates an early ontogenetic stage of the sampled individuals in the high degree of vascularity and the low degree of tissue organization. Further evidence might be the presence of globuli ossei in the caudal, indicating ongoing endochondral ossification in the caudal centrum.

In general, the high degree of cancellous bone is a feature of secondary aquatic adaptation that increased through ontogeny (i.e., bone mass decrease, see Ricqlès & Buffrénil, 2001). This fits to the observation of less compact but histologically more mature dorsal centra, when compared to the caudal centrum and the humeri. A similar pattern of ontogenetically decreasing compactness has been observed in plesiosaurs from New Zealand (Sander & Wintrich, 2021; Wiffen et al., 1995).

#### Comparison of histology and microanatomy of the two thalattosaurian taxa

In summary, *Askeptosaurus italicus* has compact bones without a medullary cavity. The tissue consists of poorly to moderately vascularized, highly organized, coarse parallel-fibred bone and extensive Sharpey’s fibers crossing those in steep angles. Because only the larger specimen (PIMUZ T 4839) shows extremely thick and coarse collagenous scaffolding of the parallel-fibred bone, we cannot exclude that this might be a specialty of this individual. However, the smaller individual (PIMUZ T 4840) also has coarse parallel-fibred tissue, although not as extreme as the larger one. Femoral, rib, and gastral tissue is stratified by growth marks in both specimens, although growth marks are less pronounced in PIMUZ T 4840.

The coarse nature and the thick fibers of the parallel-fibred bone are unique for *Askeptosaurus*. Houssaye et al., (2013) and Houssaye et al., (2014) had described the occurrence of an “unusual type of parallel-fibered bone, with large and randomly shaped osteocyte lacunae (otherwise typical of fibrous/woven bone)” for mosasaurs and ichthyosaurs, which is very different from what is observed in *Askeptosaurus*. However, one specimen of *Ichthyosaurus* (IGPB R 222) shows indeed a similar thick and coarse scaffold of the parallel-fibred tissue (NK pers. obs.).

The thalattosauroid vertebrae and humeri from the Upper Triassic of Oregon are cancellous, i.e., have a trabecular microstructure throughout, resulting in a low bone compactness. The tissue is highly vascularized and dominated by poorly organized parallel-fibred and woven matrix with only very locally restricted incipient osteonal development. The parallel-fibred tissue is also coarse but not as fibrous as that of *Askeptosaurus*. Changes in bone apposition rate and thus growth rate are indicated by changes in vascularization and organization in the Oregon thalattosauroid, no growth marks are identified.

Both, the *Askeptosaurus* material and the thalattosauroid material, share a principally radial vascular canal organization at midshaft in their long bones, although vascular canals are oriented mainly longitudinally in the smaller *Askeptosaurus* sample (PIMUZ T 4840).

All samples studied here lack periosteal lamellar bone, well developed primary osteons, and secondary osteons or Haversian bone. Woven bone is only locally identified in the humeri of the Oregon thalattosauroid. Remodeling and resorption are extensive in the cancellous bone of the Oregon material, whereas remodeling and resorption activity is much more limited in *Askeptosaurus*. The thalattosauroid from Oregon shows a high vascularity, scaffolding, and static osteogenesis of osteocytes with locally woven and poorly organized parallel-fibred tissue. However, due to the lack of primary osteons this tissue does not resemble the typical fibro-lamellar tissue type or complex. Thus, the Oregon thalattosaurian seems to shows an initial stage of fibro-lamellar complex due to a similar scaffold but differs in deposited tissue and vascular canal development.

#### Comparison with other Triassic marine reptiles

#### Vertebrae

Microstructure and vascularity of the vertebrae of *Askeptosaurus* cannot be compared due to their compressed nature*.* The high amount of secondary lamellar bone in the centrum of centra of *Askeptosaurus* might indicate an originally cancellous structure of the centra, whereas the surrounding periosteal cortex is rather compact. Vertebrae of non-plesiosaur Sauropterygia always have a large cavity or several cavities in their centra (Klein et al., 2019b). Although the vertebrae of *Askeptosaurus* are compressed, there is no evidence for the presence of large cavities such as differences or irregularities in the pattern of compaction. The amount of calcified cartilage is less in *Askeptosaurus* when compared to Sauropterygia in general (Klein et al., 2019b). The primary periosteal tissue in *Askeptosaurus* consists of coarse parallel-fibred tissue, which is not observed in vertebrae of Sauropterygia.

The centra of the thalattosauroid from Oregon differ from that of Triassic Sauropterygia by their much greater spongiosa that is evenly distributed [contrary to the spongious and compact areas in vertebrae of sauropterygians]. The centra of the thalattosaurian of Oregon lack any cavity, contrary to what is described for Sauropterygia (Klein et al., 2019b). The histology and microstructure of the plesiosaur vertebrae figured in Wintrich et al. (2017) are similar to those of other Sauropterygia and differ from both of the thalattosaurians described here by the presence of cavities within some plesiosaur centra and by the higher amount of spongiosa relative to *Askeptosaurus* and a lower amount of spongiosa relative to the Oregon thalattosauroid. The tissue in the vertebrae of the thalattosauroid from Oregon is comparable to some Eosauropterygia (Klein et al., 2016, 2019b) but differs from the placodont and plesiosaur vertebrae in the lack of fibro-lamellar tissue type (Klein et al., 2019b; Sander & Wintrich, 2021; Wintrich et al., 2020).

Ichthyosaur vertebral centra are characterized by a limited endochondral domain due to their amphicoely and disc-shape (Houssaye et al., 2018; Wintrich et al., 2020). The vertebrae of the thalattosauroid from Oregon are also disc-shaped and the endochondral domain is similarly limited as in ichthyosaurs. Ichthyosaurs and the caudal vertebra of the Oregon thalattosauroid further share a periosteal spongiosa of primary origin (Anderson et al., 2019; Houssaye et al., 2018). Vascular organization of the cortex is quite similar in the thalattosauroid and ichthyosaurs: a mixture of laminar to circumferential and radial orientation, although the radial one dominates in the thalattosauroid. The primary cancellous periosteal tissue differs: ichthyosaurs show fibrolamellar complex (Houssaye et al., 2018), whereas tissue is dominated by parallel-fibred bone in the Oregon thalattosauroid. Ichthyosaur vertebrae also differ from the *Askeptosaurus* vertebra in tissue (fibrolamellar complex vs. coarse parallel-fibred bone) and compactness (highly spongious vs. likely somewhat cancellous). To our knowledge so far, no globuli ossei have been explicitly described in any marine reptile before, although they are visible in many of the vertebrae from different amniotes sampled by Wintrich et al., (2020, supplement).

#### Ribs and gastralia

The posterior dorsal rib of *Askeptosaurus italicus* was originally oval shaped and is now compressed resulting in a very compact appearance. Whether the medullary region was originally more cancellous, i.e., contained erosion cavities can no longer be detected because of the strong compaction, but large erosion cavities (due to the overall content) and a large free medullary cavity can be excluded (i.e., due to the lack of an indication for endosteal lining of such a cavity). However, the nearly avascular cortex was also originally very compact. The ribs of other marine reptiles such as the Permian mesosaurs (Klein et al., 2019a), the Jurassic sphenodontid *Paleopleurosaurus* (Klein & Scheyer, 2017), and Triassic sauropterygians (Klein et al., 2019b; Surmik et al., 2018) are less compact when compared to the posterior dorsal rib of *Askeptosaurus italicus*, because all the others show a (also often small) free cavity. The sampled gastralium of *Askeptosaurus italicus* is similar in its poorly vascularized and dense tissue to gastralia described for eosauropterygians (Klein et al., 2019b), *Paleopleurosaurus* (Klein & Scheyer, 2017), and ichthyosaurs (Anderson et al., 2019; Kolb et al., 2011).

#### Long bones

Mesosaur femoral microanatomy and histology differs from that of the sampled thalattosaurians by a nearly avascular, highly organized, i.e., osteosclerotic tissue (Klein et al., 2019a) and that of *Paleopleurosaurus* by the presence of a large free cavity in that taxon (Klein & Scheyer, 2017). *Askeptosaurus* and some pachypleurosaurs (*Dactylosaurus*, *Neusticosaurus*, *Serpianosaurus*; Sander, 1990; Hugi et al., 2011; Klein & Griebeler, 2018) share a more compact femoral microanatomy with low to only moderate vascular density and the medullary region being filled by endosteal bone. Femora of nothosaurs and other eosauropterygians usually have an enlarged medullary cavity (Hugi, 2012; Klein & Surmik, 2021; Klein et al., 2016; Krahl et al., 2013) or show a rather reduced medullary cavity (Klein et al., 2016). However, the variability in the size of the medullary cavity in nothosaurs is clearly not a phylogenetic signal but rather depends on environment (i.e., developmental plasticity) and body size (Klein et al., 2016, 2022). Femora of *Askeptosaurus* share with most non-plesiosaur Triassic Sauropterygia the dominance of parallel-fibred tissue; however, the parallel-fibred bone in *Askeptosaurus* is unique due to its very coarse and fibrous nature (Klein, 2010; Klein & Griebeler, 2016; Klein & Surmik, 2021; Klein et al., 2015, 2016). Non-plesiosaur Sauropterygia and *Askeptosaurus* share the presence of distinct growth marks.

Vascular canal density in the smaller *Askeptosaurus* specimen is comparable to some pachypleurosaurs (*Neusticosaurus*; Klein & Griebeler, 2018), nothosaurs (Klein, 2010; Klein & Griebeler, 2016; Klein et al., 2016), and some placodonts (*Psephoderma*; Klein et al., 2015). Vascular canal density in the larger specimen of *Askeptosaurus* differs from all pachypleurosaurs (Klein, 2010; Klein & Griebeler, 2018), and is roughly comparable to some large nothosaurs (Klein et al., 2016), as well as to some placodonts (Klein et al., 2015). The femora of the larger *Askeptosaurus* and the humeri of the thalattosauroid from Oregon share a dominantly radial canal organization, which is also different from most Triassic Sauropterygia, where usually longitudinal canals dominate (pachypleurosaurs, nothosaurs), although radial ones can also dominate (some pachypleurosaurs, some placodonts, all plesiosaurs). The mixture of simple vascular canals and incompletely lined vascular canals is also shared by the two thalattosaurians and non-plesiosaur Sauropterygia. However, in thalattosauroid the canals are thinly and incompletely lined with parallel-fibred bone and not as typically well surrounded by lamellar bone.

Non-plesiosaur sauropterygians display a high amount of calcified cartilage in the medullary regions of their long bones which is only—to a minor extend—present in *Askeptosaurus* but does not occur in the thalattosauroid. The sampled thalattosaurian humeri from Oregon differ by their primary spongiosa in the outer cortex from all Triassic non-plesiosaur Sauropterygia. Some placodonts also have a radial trabecular structure but bone compactness is still higher when compared to the thalattosauroid and the tissue is fibro-lamellar complex (Klein et al., 2015). The histological features of plesiosaurs are most similar to the Oregon thalattosauroid in that they also show a radial trabecular architecture, which is, however, more compact in the former (Sander & Wintrich, 2021; Wintrich et al., 2017). In the Oregon thalattosaurian, the fibro-lamellar complex shows only an initial scaffolding.

Due to a higher compactness, lower vascular density, and overall tissue organization, femora of *Askeptosaurus* are quite different when compared to long bones of ichthyosaurs (Buffrénil & Mazin, 1990; Houssaye et al., 2014; Kolb et al., 2011). Ichthyosauria and the thalattosauroid humeri from Oregon share a primary spongious organization of the humerus (Houssaye et al., 2014; Anderson et al., 2019 but see Kolb et al., 2011 for the possible exception in mixosaurs). However, the organization of vascular canals is laminar to circumferential in ichthyosaurs (Anderson et al., 2019; Houssaye et al., 2014) but strongly radial in the thalattosaurian. As in the vertebrae, the tissue type is of the fibrolamellar complex type in ichthyosaurs but not in the thalattosaurians.

In conclusion, high bone compactness in *Askeptosaurus* resembles what is seen in some pachypleurosaurs (i.e., *Dactylosaurus*, *Neusticosaurus*, *Serpianosaurus*, *Proneusticosaurus*), and its bone tissue type fits into the variability of most non-plesiosaur eosauropterygians, except for the very coarse nature of its parallel-fibred tissue. The microanatomy of the Oregon thalattosauroid resembles what is documented in ichthyosaurs and plesiosaurs.

*Askeptosaurus* differs from all other marine reptiles by the very coarse nature and thick fibers of the parallel-fibred tissue, randomly arranged in bundles or forming a honeycomb-like pattern. The thalattosauroid from Oregon is unique due to its primary radial scaffold of parallel-fibred and woven bone but without having developed the fibro-lamellar tissue type (see definition in Francillon-Vieillot et al., 1990).

#### Thalattosaurian habitat, aquatic adaptation, and histology

Bone histology correlates with “functional” traits (Ricqlès et al., 2008), and environmental conditions seem to have a strong influence on histological traits (e.g., Klein et al., 2022; Konietzko-Meier & Klein, 2013; Sander & Klein, 2005; Teschner et al., 2022). On the other hand, a phylogenetic signal is evident at higher taxonomic levels (e.g., Klein, 2010; Ricqlès et al., 2008). Interestingly, most marine reptile groups studied to date (e.g., basal Eosauropterygia, Plesiosauria, Ichthyosauria) have an overall similar histology and microanatomy at the group level and are, therefore, distinguishable at the group level (e.g., Houssaye et al., 2014, 2018; Klein, 2010; Wintrich et al., 2017). The only exception here is the Placodontia, where some placodonts (Placodontia indet. aff. *Cyamodus*) have highly vascularized fibro-lamellar complexes, while others (*Psephoderma* and *Paraplacodus*) have very poorly vascularized lamellar tissue (Klein et al., 2015). The reason for these differences within the placodontid may be related to the different environmental conditions under which each placodont taxon lived. *Askeptosaurus italicus* and the thalattosauroid from Oregon, exhibit a similar inconsistent range of histological and microanatomical features comparable to that seen in placodonts. However, their combination of tissue, vascularization, and microanatomy clearly distinguishes them from each other, as well as from other marine reptiles.

*Askeptosaurus* and the Oregon thalattosauroid differ not only in tissue type but also in microanatomy, i.e., the compactness of the bones, suggesting different lifestyles (Ricqlès & Buffrénil, 2001). *Askeptosaurus* exhibits very compact bones (i.e., an increase in bone mass), typical of shallow-water dwellers that are in the early stages of secondary aquatic adaptation. The thalattosauroid material from Oregon shows a spongy trabecular structure (i.e., decrease in bone mass) in the humeri and vertebrae examined, which is interpreted as typical of inhabitants of open waters (pelagic) and indicates a higher degree of secondary aquatic adaptation. However, morphologically *Askeptosaurus* already shows a certain degree of aquatic adaptation, mainly referring to its strong and elongated swimming tail. Other aquatic adaptations include short limbs, broad zeugopodia, and high neural spines in the sculling tail. *Askeptosaurus* occurs in an intraplatform basin of the Alpine realm, which was at times connected to the open ocean, i.e., the Tethys. The thalattosauroid material from Oregon consists of isolated bones, and other than the paedomorphically reduced suture closure on the skull and vertebrae, not much can be said about morphological evidence for aquatic adaptation. However, the material is from a concretion found in a deep-sea marine basin, indicating open water conditions.

The differences in tissue type and microanatomy between the two thalattosaurians studied may be also related to phylogeny, as *Askeptosaurus* is a plesiomorphic thalattosaurian, whereas the Oregon fossils do represent a thalattosauroid (Druckenmiller et al., 2020; Metz, 2019).

It was previously noted that thalattosaurians did not exhibit morphological adaptations to a pelagic lifestyle (Druckenmiller et al., 2020; Rieppel et al., 2000). However, they nevertheless inhabited a range of marine settings (Druckenmiller et al., 2020; Müller, 2005). In the eastern Tethys, thalattosaurians are found in lagoonal (Sun et al., 2016) to pelagic environments (Wang et al., 2008). In North America, they occur in locations also ranging from nearshore to pelagic environments (Nicholls & Brinkmann, 1993). However, specifically thalattosauroids are often found together with ichthyosaurs that are known to have preferred open waters, as in the case of the Carnian Hosselkus Limestone of California, where the first thalattosauroids were found (Merriam, 1908), the Carnian Xiaowa Formation of Guizhou (Wang et al., 2008), and the Norian Pardonet Formation of British Columbia (Nicholls & Brinkmann, 1993). In these faunas, thalattosaurians are the only other common group besides ichthyosaurs. Interestingly, there are no thalattosaurians found in the otherwise marine reptile-rich deposits of the nearshore and shallow marine environments of the Germanic Basin (i.e., the Muschelkalk deposits). The differences in microanatomy between *Askeptosaurus* and the Oregon taxon could, therefore, be due to differences in environment and lifestyle, i.e., the Oregon thalattosaurian may have lived in more open waters than *Askeptosaurus*.

#### Globuli ossei

The presence of globuli ossei in the vertebrae of the Oregon thalattosauroid is noteworthy. Globuli ossei represent chondrocyte lacunae secondarily filled by endosteal bone deposits e.g., Francillon-Vieillot et al., 1990; Buffrénil et al., 2014; Quilhac et al., 2014, Buffrenil & Quilhac 2021. According to previous studies (e.g., Beresford, 1981, pp. 358–360), globuli ossei are taxonomically relatively common among various bone elements in tetrapods, but are rarely described in vertebrae and long bones. They have previously been documented in long bones of amphibians (Quilhac et al., 2014), an extinct bird (Marsà et al., 2018), and in an extinct whale from the Late Eocene Buffrénil et al., 2021a. According to Ricqlès & Buffrénil (2001), osteosclerosis in sirenians and *Basilosaurus* is the result of an endochondral sequence that stops at the “globuli ossei” stage. The presence of globuli ossei in the thalattosauroid centrum from Oregon is, to our knowledge, the first explicit description of this structure in a marine reptile (but see supplement of Wintrich et al. (2020).

The occurrence of globuli ossei in the shaft of postcranial bones of tetrapods at relatively advanced ontogenetic stages has been associated with a delay or inhibition of endochondral osteogenesis (Buffrénil et al., 1990). This is supported by the findings of Quilhac et al. (2014) in the long bones of postmetamorphic urodeles. These authors also concluded that globuli ossei are associated with a low rate of length growth (Quilhac et al., 2014). The reduction in endochondral osteogenesis has also been associated with an increase in bone mass and osteosclerosis in aquatic vertebrates and interpreted as a secondary adaptation to aquatic life (Buffrénil et al., 1990).

The presence of globuli ossei in an extinct penguin is according to the authors not related to an adaptation to aquatic life, small body size, ontogenetic stage, or a pathology (Marsà et al., 2018). They suggest more caution when interpreting histological structures and the animal’s way of life and an investigation on the entire ossification process of the respective bones that could provide insights on the nature of the globuli ossei. Because the globuli ossei are part of the endochondral ossification below the articular surface, their presence here in the caudal centrum of the Oregon thalattosaurian is not unusual. What is unexpected, however, is the delayed ossification in this already relatively large animal.

## Conclusions

Microanatomy (i.e., bone compactness) and tissue type of the two thalattosaurian taxa studied followed different growth rates and patterns and thus different life history strategies. *Askeptosaurus* from the late Anisian of Monte San Gorgio exhibits high bone compactness, indicating an increase in bone mass. Its bone tissue indicates relatively slow to moderate growth rates. The thalattosauroid from the late Carnian of the Vester Formation in Oregon shows a primary trabecular (radial) tissue organization and thus bone mass decrease. Its tissue type implies much higher growth rates when compared to *Askeptosaurus* due to lower organization and higher vascularity of tissue. Further on, histology (i.e., presence of globuli ossei) of the thalattosauroid from Oregon seems to indicate evidence of pedomorphosis.

Such marked differences within a group are rather atypical for Triassic marine reptiles (i.e., Eosauropterygia including plesiosaurs, Ichthyosauria), as marine reptiles at the group level generally share the same general bone histology, i.e., growth rates and patterns. The only other exception, where histology and microanatomy are very different between two taxa of the same clade is found in the placodonts. As for placodonts, environmental differences might be a possible explanation for these distinct differences in histology and microanatomy between the two thalattosaurians.

Although the bone histology and microanatomy of *Askeptosaurus italicus* is in general comparable to that of some eosauropterygians (i.e., tissue, moderate to slow vascular density, compactness), it differs in some aspects (i.e., very coarse nature of parallel-fibred tissue, strong radial arrangement of vascular canals). This once more demonstrates that differences in growth rate, and growth patterns, implying differences in lifestyle and life history traits, allowed all of these Triassic marine reptiles to live simultaneously in the same environments (i.e., shallow marine intraplatform basins and epicontinental seas) in the Tethys without significant competition.

The bone histology of the Oregon thalattosauroid largely resembles fast growth rates of ichthyosaurs and plesiosaurs (radial vascular pattern, quickly deposited tissue, scaffold architecture) but without having deposited true fibro-lamellar complex.

## Data Availability

All samples studied here are stored at the MNCH, University of Oregon Museum of Natural and Cultural History, Eugene, Oregon, and the Palaeontological Institute and Museum, University of Zurich, Zurich, according to their repository number (see Table 1).

## Abbreviations

MNCH: University of Oregon Museum of Natural and Cultural History, Eugene, Oregon
PIMUZ: Paleontological Institute and Museum, University of Zurich, Zurich, Switzerland

## References

[CR1] Anderson KL, Druckenmiller PS, Erickson E, Maxwell EE (2019). Skeletal microstructure of *Stenopterygius quadriscissus* (reptilia, ichthyosauria) from the posidonienschiefer (posidonia shale, Lower Jurassic) of Germany. Paleontology.

[CR2] Bakker RT, Thomas DK, Olson EC (1980). Dinosaur heresy-dinosaur renaissance: why we need endothermic archosaurs for a comprehensive theory of bioenergetic evolution. A cold look at warm-blooded dinosaurs.

[CR87] Beresford, W. A. 1981. Chondroid bone, secondary cartilage and metaplasia. Munich: Urban and Schwarzemberg.

[CR3] Buffrénil, V. de, Zylberberg X, Ricqlès, A. de, Padian, K. (Eds.) (2021b).* Vertebrate Skeletal Histology and Paleohistology*. Boca Raton: CRC Press.

[CR4] Buffrénil, V. de, & Mazin, J.-M. (1990). Bone histology of the ichthyosaurs: comparative data and functional interpretation. *Paleobiology,**16*, 435–447. 10.1017/S0094837300010174

[CR7] de Buffrénil V, Castanet J (2000). Age estimation by skeletochronology in the nile monitor lizard (*Varanus niloticus*), a highly exploited species. Journal of Herpetology.

[CR8] de Buffrénil V, de Ricqlès A, Ray CE, Domning DP (1990). Bone histology of the ribs of the archaeocetes (mammalia: cetacea). Journal of Vertebrate Paleontology.

[CR9] de Buffrénil V, Laurin M, Jouve St, de Buffrénil V, Zylberberg X, de Ricqlès A, Padian K (2021). Archosauromorpha: the crocodylomorpha. Vertebrate skeletal histology and paleohistology.

[CR10] de Buffrénil V, Quilhac A, de Buffrénil V, Zylberberg X, de Ricqlès A, Padian K (2021). Bone tissue types: a brief account of currently used categories. Vertebrate skeletal histology and paleohistology.

[CR86] Buffrénil, V. de, Aurore Canoville, Susan E. Evans & Michel Laurin (2015) Histological study of karaurids, the oldest known (stem) urodeles. *Historical Biology*, *27*(1), 109–114. 10.1080/08912963.2013.869800

[CR5] Canoville A, Laurin M (2010). Evolution of humeral microanatomy and lifestyle in amniotes, and some comments on palaeobiological inferences. Biological Journal of the Linnean Society.

[CR15] Druckenmiller PS, Kelley NP, Metz ET, Baichtal J (2020). An articulated late triassic (Norian) thalattosauroid from Alaska and ecomorphology and extinction of Thalattosauria. Scientific Reports.

[CR16] Dumont M, Laurin M, Jaques F, Pellé E, Dabin W, de Buffrénil V (2013). Inner architecture of vertebral centra in terrestrial and aquatic mammals: a two-dimensional comparative study. Journal of Morphology.

[CR17] Francillon-Vieillot H, de Buffrénil V, Castanet J, Géraudie J, Meunier FJ, Sire JY, Zylberberg L, de Ricqlès A, Carter JG (1990). Microstructure and mineralization of vertebrate skeletal tissues. Skeletal biomineralization: patterns, processes and evolutionary trends.

[CR18] Furrer, H. (2003). Der Monte San Giorgio im Südtessin - Vom Berg der Saurier zur Fossil-Lagerstätte internationaler Bedeutung. Neujahrsblatt, Naturforschende Gesellschaft in Zürich [Njbl. natf. Ges. Zürich] 206: 1–64

[CR19] Furrer H (1995). The prosanto formation, a marine middle triassic fossil-lagerstätte near davos (canton graubünden, eastern swiss alps). Eclogae Geologicae Helvetiae.

[CR20] Griebeler E-M, Klein N (2019). Life-history strategies indicate live-bearing in *Nothosaurus* (Sauropterygia). Palaeontology.

[CR21] Houssaye A, Lindgren J, Pellegrini R, Lee AH, Germain D, Polcyn MJ (2013). Microanatomical and histological features in the long bones of mosasaurine mosasaurs (reptilia, squamata)—implications for aquatic adaptation and growth rates. PLoS ONE.

[CR22] Houssaye A, Mazurier A, Herrel A, Volpato V, Taffereou P, Boistel R, de Buffrénil V (2010). Vertebral microanatomy in squamates: structure, growth, and ecological correlates. Journal of Anatomy.

[CR23] Houssaye A, Nakajima Y, Sander PM (2018). Structural, functional, and physiological signals in ichthyosaur vertebral centrum microanatomy and histology. Geodiversitas.

[CR24] Houssaye A, Sander PM, Klein N (2016). Adaptive patterns in aquatic amniote bone microanatomy—more complex than previously thought. Integrative & Comparative Biology.

[CR25] Houssaye A, Scheyer TM, Kolb C, Fischer V, Sander PM (2014). A new look at ichthyosaur long bone microanatomy and histology: implications for their adaptation to an aquatic life. PLoS ONE.

[CR27] Hugi J (2012). The long bone histology of *Ceresiosaurus* (sauropterygia, reptilia) in comparison to other eosauropterygians from the Middle Triassic of Monte San Giorgio. Swiss Journal of Palaeontology.

[CR28] Hugi J, Scheyer TM, Sander PM, Klein N, Sánchez-Villagra MR (2011). Long bone microstructure gives new insights into the life history data of pachypleurosaurids from the middle triassic of monte san giorgio, Switzerland/Italy. Comptes Rendus Palevol.

[CR29] Jaquier VP, Scheyer TM (2017). Bone histology of the middle triassic long-necked reptiles *Tanystropheus* and *Macrocnemus* (archosauromorpha, Protorosauria). Journal of Vertebrate Paleontology.

[CR30] Klein N (2010). Long bone histology of Sauropterygia from the Lower Muschelkalk of the Germanic Basin provides unexpected implications for phylogeny. PLoS ONE.

[CR31] Klein N, Canoville A, Houssaye A (2019). Microstructure of vertebrae, ribs, and gastralia of Triassic sauropterygians—new insights into the microanatomical processes involved in aquatic adaptations of marine reptiles. The Anatomical Record.

[CR32] Klein N, Eggmaier ST, Hagdorn H (2022). The redescription of the holotype of Nothosaurus mirabilis (Diapsida, Eosauropterygia)—a historical skeleton from the Muschelkalk (Anisian, Middle Triassic) near Bayreuth (southern Germany). PeerJ.

[CR33] Klein N, Griebeler E-M (2016). Bone histology, microanatomy, and growth of the nothosauroid *Simosaurus gaillardoti* (sauropterygia) from the upper muschelkalk of southern Germany/Baden-Württemberg. Comptes Rendus Palevol.

[CR34] Klein N, Griebeler E-M (2018). Growth patterns, sexual dimorphism, and maturation modelled in pachypleurosauria from middle triassic of central Europe (diapsida: sauropterygia). Fossil Record.

[CR35] Klein N, Houssaye A, Neenan JM, Scheyer TM (2015). Long bone histology and microanatomy of Placodontia (Diapsida: Sauropterygia). Contributions to Zoology.

[CR36] Klein N, Sander PM (2007). Bone histology and growth of the prosauropod *Plateosaurus engelhardti* meyer, 1837 from the Norian bonebed of trossingen (Germany) and frick (Switzerland). Special Papers in Paleontology.

[CR37] Klein N, Sander PM, Krahl A, Scheyer TM, Houssaye A (2016). Diverse aquatic adaptations in *Nothosaurus* spp. (Sau-rop-tery-gia)—inferences from humeral histology and microanatomy. PLoS ONE.

[CR38] Klein N, Sander PM, Stein K, Le Loeuff J, Carballido JL, Buffetaut E (2012). Modified laminar bone in *Ampelosaurus atacis* and other titanosaurs (Sauropoda): implications for life history and physiology. PLoS ONE.

[CR39] Klein N, Scheyer TM (2017). Microanatomy and life history in *Palaeopleurosaurus* (rhynchocephalia: pleurosauridae) from the early jurassic of Germany. The Science of Nature.

[CR40] Klein N, Surmik D (2021). Bone histology of eosauropterygian diapsid *Proneusticosaurus silesiacus* from the Middle triassic of Poland reveals new insights into taxonomic affinities. Acta Palaeontologica Polonica.

[CR41] Klein N, Verrière A, Satorelli H, Wintrich T, Fröbisch J (2019). Microanatomy and growth record of *Stereosternum tumidum* cope 1886 (Sauropsida, Parareptilia). Fossil Record.

[CR42] Köhler M, Marín-Moratalla N, Jordana X, Aanes R (2012). Seasonal bone growth and physiology in endotherms shed light on dinosaur physiology. Nature.

[CR43] Kolb C, Sánchez-Villagra MR, Scheyer TM (2011). The palaeohistology of the basal ichthyosaur *Mixosaurus* (Ichthyopterygia, Mixosauridae) from the Middle Triassic: Palaeobiological implications. Comptes Rendus Palevol.

[CR44] Konietzko-Meier D, Klein N (2013). Unique growth pattern of *Metoposaurus diagnosticus* (Amphibia, Temnospondyli) from the Upper Triassic of Krasiejów. Palaeogeography, Palaeoclimatology, Palaeoecology.

[CR45] Konietzko-Meier D, Sander PM (2013). Long bone histology of *Metoposaurus diagnosticus* (Temnospondyli) from the Late Triassic of Krasiejów (Poland) and its paleobiological implications. Journal of Vertebrate Paleontology.

[CR46] Krahl A, Klein N, Sander PM (2013). Evolutionary implications of the divergent long bone histologies of *Nothosaurus* and *Pistosaurus* (Pis-to-saur-us, Triassic). BMC Evolutionary Biology.

[CR47] Kuhn E (1952). Die Triasfauna der Tessiner Kalkalpen. XVII: *Askeptosaurus italicus* Nopsca. Schweizer Paläontologische Abhandlungen.

[CR48] Kuhn-Schnyder E (1960). Über einen Schultergürtel von *Askeptosaurus italicus* Nopsca aus der anisischen Stufe der Trias des Monte San Giorgio (Kt. Tessin, Schweiz). Eclogae Geologicae Helvetiae.

[CR49] Kuhn-Schnyder E (1971). Über einen Schädel von *Askeptosaurus italicus* Nopsca aus der mittleren Trias des Monte San Giorgio (Kt. Tessin, Schweiz). Abhandlungen Des Hessischen Landesamts Für Bodenforschung.

[CR50] Marsà JAG, Tambussi CP, Cerda IA (2018). First evidence of globuli ossei in birds (Aves, Spheniciformes). Implications on paleohistology and bird behaviour. Historical Biology.

[CR85] Merriam, J. C. 1908. Triassic Ichthyosauria, with special reference to the American forms. Memoirs of the University of California, 1, 1–155.

[CR51] Metz, E. T. (2019).* Description, phylogenetic analysis and taphonomy of a new thalattosaur from the Brisbois Member of the Vester Formation (Carnian/Norian) of Central Oregon*. Thesis (MSc Thesis) University of Alaska Fairbanks, 2019. http://hdl.handle.net/11122/10519.

[CR52] Montes L, Le Roy N, Perret M, de Buffrenil V, Castanet J, Cubo J (2007). Relationships between bone growth rate, body mass and resting metabolic rate in growing amniotes: a phylogenetic approach. Biological Journal of the Linnean Society.

[CR53] Müller, J. (2002). *A revision of Askeptosaurus italicus and other thalattosaurs from the European Triassic, the interrelationships of thalattosaurs, and their phylogenetic position within diapsid reptiles (Amniota, Eureptilia).* PhD thesis, Johannes-Gutenberg-Universität, Mainz, 208 pp. https://openscience.ub.uni-mainz.de/handle/20.500.12030/3940

[CR54] Müller J (2005). The anatomy of *Askeptosaurus italicus* from the Middle Triassic of Monte San Giorgio and the interrelationships of thalattosaurs (Reptilia, Diapsida). Canadian Journal of Earth Sciences.

[CR55] Müller J, Renesto S, Evans SE (2005). The marine diapsid reptile *Endennasaurus* (Reptilia: Thalattosauriformes) from the Late Triassic of Italy. Palaeontology.

[CR56] Nakajima Y, Hirayama R, Endo H (2014). Turtle humeral microanatomy and its relationship to lifestyle. Biological Journal of the Linnean Society.

[CR57] Nicholls EL, Brinkman D (1993). New thalattosaurs (Reptilia: Diapsida) from the Triassic Sulphur Mountain formation of Wapiti Lake, British Columbia. Journal of Paleontology.

[CR78] von Nopsca F (1925). *Askeptosaurus*, ein neues Reptil aus der Trias von Besano. Centralblatt Für Mineralogie, Geologie Und Paläontologie.

[CR58] Padian K, Horner JR, Weishampel D, Dodson P, Osmolska H (2004). Physiology. The dinosauria.

[CR59] Padian K, Horner JR, de Ricqlès A (2004). Growth in small dinosaurs and pterosaurs: the evolution of archosaurian growth strategies. Journal of Vertebrate Paleontology.

[CR61] Quémeneur S, de Buffrénil V, Laurin M (2013). Microanatomy of the amniote femur and inference of lifestyle in limbed vertebrates. Biological Journal of the Linnean Society.

[CR62] Quilhac A, de Ricqlès A, Lamrous H, Zylberberg L (2014). Globuli ossei in the long limb bones of *Pleurodeles waltl* (Amphibia, Urodela, Salamandridae). Journal of Morphology.

[CR11] de Ricqlès A (1983). Cyclical growth in the long limb bones of a sauropod dinosaur. Acta Palaeontologica Polonica.

[CR12] de Ricqlès A, Adler K (1992). Paleoherpetology now: a point of view. Herpelotogy: current reseach on the biology of amphibians and reptiles, proceedings: first world herpetology congress.

[CR13] de Ricqlès A, de Buffrénil V, Mazin JM, de Buffrénil V (2001). Bone histology, heterochronies and the return of tetrapods to life in water: where are we?. Secondary adaptations of tetrapods to life in water.

[CR14] de Ricqlès A, Padian K, Knoll F, Horner JA (2008). On the origin of high growth rates in archosaurs and their ancient relatives: complementary histological studies on triassic archosauriforms and the problem of a “phylogenetic signal” in bone histology. Annales De Paleontologie.

[CR63] Rieppel O (1987). *Clarazia* and *Hescheleria*: a re-investigation of two problematical reptiles from the middle triassic of Monte San Giorgio (Switzerland). Palaeontographica A.

[CR64] Rieppel O, Liu J, Bucher H (2000). The first record of a thalattosaur reptile from the late triassic of Southern China (Guizhou Province, PR China). Journal of Vertebrate Paleontology.

[CR65] Rieppel O, Müller J, Liu J (2005). Rostral structure in Thalattosauria (Reptilia, Diapsida). Canadian Journal of Earth Sciences.

[CR66] Sander PM (1990). Skeletochronology in the small Triassic reptile *Neusticosaurus*. Annales Des Sciences Naturelles.

[CR67] Sander, P. M. (2021). Ichthyosauria. In Buffrénil, V. de, X. Zylberberg, A., Ricqlès, A. de & K. Padian (Eds.), *Vertebrate skeletal histology and paleohistology* (pp. 458–466). CRC Press.

[CR68] Sander PM, Klein N (2005). Unexpected developmental plasticity in the life history of an early dinosaur. Science.

[CR83] Sander, P. M. (2000). Long bone histology of the Tendaguru sauropods: Implications for growth and biology. *Paleobiology, 26*, 466–488.

[CR69] Sander, P. M., & Wintrich, T. (2021). Plesiosauria. In Buffrénil, V. de, X. Zylberberg, Ricqlès, A. de & K. Padian (Eds.), *Vertebrate skeletal histology and paleohistology* (pp. 444–457). CRC Press.

[CR70] Scheyer MT, Houssaye A, Klein N, de Buffrénil V, Zylberberg X, de Ricqlès A, Padian K (2021). Nothosaurs and pachypleurosaurs. Vertebrate skeletal histology and paleohistology.

[CR71] Scheyer MT, Klein N, de Buffrénil V, Zylberberg X, de Ricqlès A, Padian K (2021). Placodontia. Vertebrate skeletal histology and paleohistology.

[CR73] Scheyer TM, Neenan JM, Bodogan T, Furrer H, Obrist CH, Plamondon M (2017). A new, exceptionally preserved juvenile specimen of *Eusaurosphargis dalsassoi* (Diapsida) and implications for mesozoic marine diapsid phylogeny. Scientific Reports.

[CR74] Spiekman SNF, Neenan JM, Fraser NC, Fernandez V, Rieppel O, Nosotti S, Scheyer TM (2020). Aquatic habits and niche partitioning in the extraordinarily long-necked Triassic reptile *Tanystropheus*. Current Biology.

[CR75] Sun Z, Jiang D, Ji Ch, Hao W (2016). Integrated biochronology for Triassic marine vertebrate faunas of Guizhou Province, South China. Journal of Asian Earth Sciences.

[CR76] Surmik D, Szczygielski T, Janiszewska K, Rothschild BM (2018). Tuberculosis-like respiratory infection in 245-million-year-old marine reptile suggested by bone pathologies. Royal Society Open Sciences.

[CR77] Teschner E, Konietzko-Meier D, Klein N (2022). Growth and limb bone histology of aetosaurs and phytosaurs from the Late Triassic Krasiejów locality (SW Poland) reveals strong environmental influence on growth pattern. Contributions to Zoology.

[CR79] White CR (2011). Allometric estimation of metabolic rates in animals. Comparative Biochemistry and Physiology, Part A.

[CR80] Wiffen J, de Buffrénil V, de Ricqles A, Mazin J-M (1995). Ontogenetic evolution of bone structure in Late Cretaceous Plesiosauria from New Zealand. Geobios.

[CR81] Wintrich T, Scaal M, Böhmer C, Schellhorn R, Kogan I, Reest A, Sander MP (2020). Palaeontological evidence reveals convergent evolution of intervertebral joint types in amniotes. Science and Reports.

[CR82] Wintrich T, Scaal M, Sander PM (2017). Foramina in plesiosaur cervical centra indicate a specialized vascular system. Fossil Record.

[CR84] Xiaofeng, W., Bachmann, G. H., Hagdorn, H., Sander, P.M., Cuny, G., Xiaohong, C., Chuanshang, W., Lide, C., Long, C., Fansong, M., Guanghong, X. (2008). The Late Triassic black shales of the Guanling area. *Guizhou Province*, south‐west China: a unique marine reptile and pelagic crinoid fossil Lagerstätte. *Palaeontology*. *51*(1):27–61.

